# Socio-cultural implications for women’s menstrual health in the Pacific Island Countries and Territories (PICTs): a scoping review

**DOI:** 10.1186/s12978-022-01398-7

**Published:** 2022-06-02

**Authors:** Elizabeth Maulingin-Gumbaketi, Sarah Larkins, Maxine Whittaker, Gun Rembeck, Ronny Gunnarsson, Michelle Redman-MacLaren

**Affiliations:** 1 College of Medicine and Dentistry, James Cook University, Townsville, QLD Australia; 2 College of Public Health, Medical and Veterinary Sciences, James Cook University, Townsville, QLD Australia; 3 Research, Education, Development & Innovation, Primary Health Care, Alsvsborg Region, Västra Götaland, Sweden; 4 General Practice/Family Medicine, School of Public Health and Community Medicine, Institute of Medicine, Sahlgrenska Academy, University of Gothenburg, Göteborg, Sweden; 5 Regionhälsan Borås Youth Health Center, Vastra Gotaland, Sweden; 6 Primary Health Care Clinic for Homeless People, Närhälsan, the Västra Götaland Region, Göteborg, Sweden

**Keywords:** Menarche, Menstruation, Menstrual health and hygiene, Socio-cultural norms, Practices females, Pacific Island Countries and Territories

## Abstract

**Background:**

Globally, experiences of menarche and subsequent menstruation are embedded in social and cultural beliefs, norms and practices. Menarche is an important developmental milestone in sexual and reproductive health (SRH) for females. Menarche is intertwined with socio-cultural norms, beliefs and practices, which can impact on women’s ability to manage menstruation with dignity. This paper reviews the social and cultural factors that affect women’s ability to effectively manage their menstrual health and hygiene (MHH) in Pacific Island Countries and Territories (PICTs).

**Methods:**

A scoping review was conducted following PRISMA scoping review guidelines and inclusion/exclusion criteria. An online search was conducted for peer-reviewed publications in Medline/OVID; Medline/PubMED; PsycINFO; CINAHL; Scopus and JSTOR, and Google Scholar. A search for grey literature was conducted in Google Scholar and websites of international and local organizations. Experts in the field also contributed additional references. Extracted data were summarised in an Excel spreadsheet. Searches were conducted between May and June, 2019, and then repeated in July, 2020.

**Results:**

A total of 11 studies were included; 10 qualitative and one mixed methods study. Studies were conducted in Melanesian (n = 9), Polynesian (n = 1) and Micronesian (n = 1) PICTs. All 11 studies reported elements of societal and personal factors; ten studies reported evidence relating to interpersonal factors; nine studies reported elements relating to environmental factors; and two studies presented evidence linked to biological factors. Managing menstrual health with dignity is challenging for many women and girls because menstruation is associated with menstrual taboos and shame.

**Conclusion:**

This review found that the MHH experiences of women in PICTs are affected by social and cultural beliefs, norms and practices. Beliefs, norms and practices about menarche need to be incorporated in SRH planning, programs and education in order to be relevant to diverse village and urban settings.

## Plain language Summary

Globally, menarche is an important developmental milestone for females. Experiences of menarche and subsequent menstruation are embedded in socio-cultural norms and practices which can impact women’s ability to manage menstruation with dignity. This paper reviews social and cultural factors that affect the ability of women to effectively manage their menstrual health and hygiene (MHH) in Pacific Island Countries and Territories (PICTs). Peer-reviewed and grey literature were searched systematically, using the PRISMA extended scoping review methodology. Eleven studies met the inclusion and exclusion criteria: 10 qualitative studies and one mixed methods study. Included studies had been conducted in Melanesian (n = 9), Polynesian (n = 1) and Micronesian (n = 1) states of PICTs. Factors affecting MHH were identified and categorised using the Socio-Ecological Framework categories: biological; personal; interpersonal; environmental and societal. All 11 studies reported elements of societal and personal factors; ten studies reported evidence relating to interpersonal factors; nine studies reported elements relating to environmental factors; and two studies presented evidence linked to biological factors. MHH experiences of women in PICTs are shaped by socio-cultural norms and practices. These norms and practices need to be incorporated into sexual and reproductive health planning, programs and education in order to be relevant to diverse village and urban settings in PICTs.

## Background

Menstrual health from menarche (first menstruation) is a girl’s right [1–3]. However, it is increasingly recognised in development and academic fields that the experiences of girls at menarche are inextricably linked to social and cultural norms, beliefs and practices. Menarche in pubertal girls signifies sexual and reproductive maturation [4], and menstrual health is an important aspect of the broader outcomes of sexual and reproductive health and rights [1–3, 5]. We use ‘girls’ to refer to pre-menarcheal females and ‘women’ to refer to both females at menarche and during their reproductive life, reflecting a common change in the social status of girls to womanhood at menarche [4, 6, 7].

“Menstrual health is a state of complete physical, mental, and social well-being and not merely the absence of disease or infirmity, in relation to the menstrual cycle” [8–10, p. 2]. This definition incorporates the need for access to accurate menstrual health information: access to timely diagnosis; treatment and care resulting from menstrual discomfort and disorders; access to Water, Sanitation and Hygiene (WASH) facilities and products for managing menstruation; positive and respectful environment that is free from stigma and discrimination; and to participate in all spheres of life during menstrual cycles without exclusion and discrimination [8–10]. Managing menstrual health with adequate knowledge, safety and dignity, without stigma is a human right for women [1–3].

Given the significance of menarche and menstruation in women’s sexual and reproductive biology, both these fundamental events are closely associated with socio-cultural norms and practices in many Low and Middle Income Countries (LMICs) [7, 11, 12]. Many of these norms and practices involve perceptions of menstrual blood as being dirty and polluting, associated with restrictive practices and stigma such as shaming and social exclusion for women during menstruation [13–15]. Studies in LMICs have found significant associations between cultural beliefs and practices and the impact on women’s MHH [2, 11, 16, 17]. However, the extent of the impact of these beliefs and practices on women’s ability to manage MHH are contextual and varied [2, 11]. There is a need for more context specific understanding of socio-cultural norms, beliefs and practices about menarche and menstruation to understand how best to address women’s menstrual health and wellbeing.

Pacific Island Countries and Territories (PICTs) in the South Pacific region comprise Polynesian, Melanesian and Micronesian countries with diverse cultural beliefs, norms and practices. Menarche is an important cultural event in most cultures in the region [15, 18]. For example, menarche is associated with restrictive practices based on the perception that menstrual blood is harmful, consequently affecting girl’s and women’s menstrual health [15, 18, 19]. However, the beliefs and practices around menarche and menstruation differ between countries and cultures [11, 20]. Beyond the anthropological literature, more evidence is required about the impact of socio-cultural norms, beliefs and practices on women’s MHH in PICTs. These studies have started to emerge in the past decade with findings that cultural norms limit women’s ability to manage menstrual health with dignity [15]. However, there is a need for more comprehensive understanding of the impact of socio-cultural beliefs, norms and practices on MHH at the regional level.

## Aim and objectives of scoping review

A scoping review was conducted to evaluate, analyse and document existing evidence about the social and cultural norms, beliefs and practices about menstruation including menarche, and their implication on women’s health and wellbeing in PICTs.

## Methods

This scoping review was performed using the PRISMA extension for scoping reviews [21]. This approach is suitable for questions where literature uses various approaches and methodologies and is found in both peer-reviewed and grey literature. The search strategy was designed to identify peer-reviewed scholarly publications and unpublished grey literature about the experiences of women at menarche and during menstruation in PICTs.

### Identifying publications

Searches for peer-reviewed and grey literature were done initially between May and June, 2019, and then repeated in July, 2020 using the same search strategy with the aim of capturing any additional literature. A librarian assisted in the design of the search strategy and guided the process of extracting data. The initial search was conducted on Medline/PubMED to modify and confirm the search terms.

### Search terms

Search terms included relevant Medical Subject Headings (MeSH) terms corresponding to these search terms in each of the databases that had thesauri (Table 1). These search terms were identified to meet the aim of the review.

**Table 1:** Standard search terms

Concept number	Concepts	Search terms
1	Menarche and menstruation	Menarche OR Menarc* OR Menstrua* OR Menses*
		AND
2	Social and cultural beliefs and practices	Social* OR Cultur* OR Custom* OR Belief* OR Folk* OR Ceremon* OR Taboo* OR Practic* OR Tradition*
		AND
3	Women’s health and wellbeing	Wom#n OR menstrua* OR health* OR hygiene* OR wellbeing*
		AND
4	Pacific Island Countries and Territories	Pacific* OR Melanesia* OR Micronesia* OR Polynesia* OR Samoa* OR Cook* or Fiji* OR Guam* OR Kiribati* OR Marshall* OR Nauru* OR Caledonia* OR Niue* OR Mariana* OR Palau* OR "Papua New Guinea" OR Solomon* OR Tokelau* OR Tonga* OR Tuvalu* OR Vanuatu* OR "Wallis Futuna"

Standard search terms were applied as per the review protocol for Google Scholar and institutional websites. However, within Google Scholar there were word limits of approximately 32 words (excludes connectors such as “AND” and “OR”)) to the search strings [22]. In such situation, the concept with the longest search string (Concept 4) was divided into six parts with connector AND)) to search terms of concept 3 and connector OR)) thereafter until all search terms of concept 4 were completed. While the limitations of Google Scholar are widely known [22–24], due to timing constraints we limited the results to the first 100 returns per search. Altogether, six searches were done resulting in 600 relevant hits, which were all downloaded and exported into Endnote™ reference manager. For the websites, where there were functions that allowed search strings to be entered, full search terms were entered. In situations where the search strings were lengthy and could not be accommodated, a number of searches were done. Smaller search functions, minimum search terms containing key concepts such as ‘((Menstruation OR Menarche AND Culture AND Women OR Health OR Hygiene AND Pacific))’ were used. All hits were scanned and 35 relevant articles retrieved. Relevant articles were manually downloaded and systematically grouped for analysis.

### Peer-reviewed search strategy

Upon confirmation of the search terms, an extensive search was performed for peer-reviewed literature in six databases: Medline/OVID; Medline/PubMED; PsycINFO; CINAHL; Scopus and JSTOR. All relevant hits were exported to Endnote library management software. Based on the functionality of Google Scholar [22], some peer-reviewed literature not successfully retrieved from databases were retrieved from this source.

### Expert search strategy

Experts working in the field relating to MHH in various capacities were contacted for additional references. This resulted in another 16 references gathered and screened.

### Grey literature search strategy

Grey literature was searched on the Google Scholar database and twenty-eight relevant websites of international and local organizations dealing with menstrual issues. Grey literature refers to “information produced at all levels of government, academic, business and industry in electronic and print formats not controlled by commercial publishing i.e. where publishing is not the primary activity of the producing body” [25, p. 70]. This includes evidence such as theses, organizational and government reports, policy papers, and conference proceedings [22, 25, 26].

### Inclusion and exclusion criteria

The following inclusion and exclusion criteria were applied consistently throughout the search and analysis stage to find relevant literature that addressed the research question [27]. To capture documents about socio-cultural norms, beliefs and practices around menstruation and their implications for women’s health and wellbeing in PICTs, we included peer-reviewed and grey literature according to the criteria shown below;

1. Examined women’s experiences around menarche and menstruation in relation to Menstrual Health and Hygiene (MHH) in PICTs.
2. Were published between 1979 and 2020 (year of the review)-1979 was the year when UN General Assembly adopted the Convention on the Elimination of All Forms of Discrimination against Women [28].
3. Examined socio-cultural norms, beliefs and practices around menstruation and their implications for women’s MHH in PICTs.
4. Examined experiences of women who were born, raised and had menarche in PICTs.
5. Studies conducted in PICTs, or reflecting on PICTs.
6. Studies conducted in English language.

Exclusion criteria

1. Studies involving PICTs migrants onto other countries.
2. Studies examining experiences of women with disabilities in PICTs.
3. Anthropological studies that lacked discussion about MHH.

### Screening and selection of articles

**Fig. 1:**
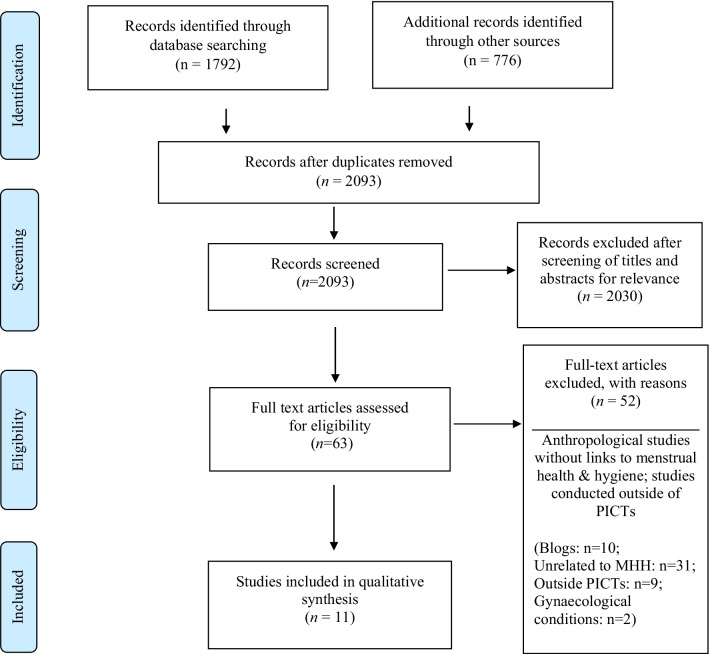
PRISMA flow diagram

A total of 2,568 articles were found with the initial search and were included in the initial screening by abstract and title (Fig. 1).

The first four phases of the PRISMA process were used to screen articles identified from databases and other sources [21, 29]. A total of 2093 articles were included after duplicates were removed. In the screening phase, title and abstract screening were done by the first author resulting in 63 eligible articles for full text review. Fifty-two articles were excluded with reasons (Fig. 1). All blogs were excluded after quality assessment for lacking methodological rigour. Full text screening was conducted independently by two authors (EMG and MRM) to determine the final articles for inclusion [21, 29]. The two authors later discussed the findings to determine which articles to include or exclude based on the inclusion criteria, with support from Author Two (SL). This process resulted in 11 articles meeting eligibility for qualitative synthesis.

Group discussion was necessary to resolve differences raised in assessments of full text of articles [21, 29, 30]. After the final list of articles for inclusion was agreed, the lead author reviewed the articles and extracted relevant data with cross-checking by other authors. The articles included were original peer-reviewed research articles, research reports, review papers, program descriptions and reports, policy papers, discussion papers and commentaries.

## Quality assessment and characteristics of articles

The Critical Appraisal Skills Program (CASP) quality assessment tool was used to assess the rigour, credibility and relevance of included articles using the relevant CASP assessment checklist [31]. Quantitative and qualitative CASP checklists were used in this assessment; ten studies were qualitative and one was quantitative (Table 3). No articles were excluded on quality grounds at this stage.

## Data extraction and analysis

**Fig. 2:**
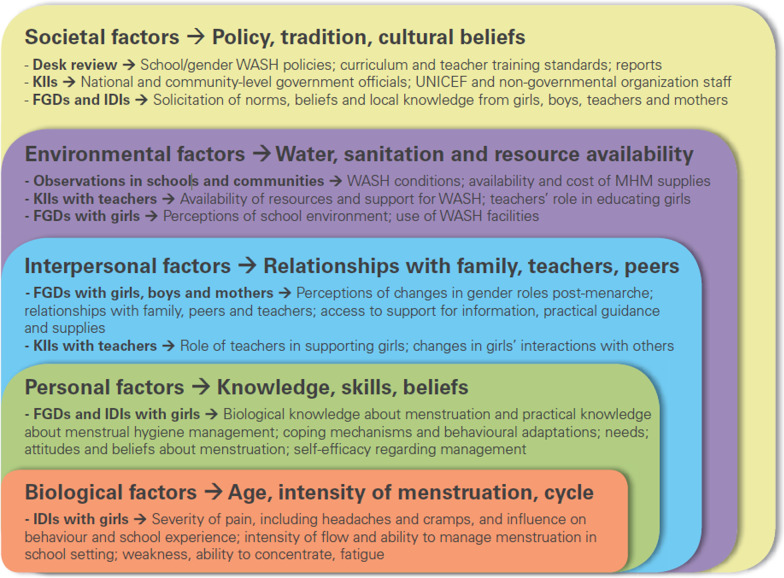
Socio-ecological framework for menstrual hygiene management. *Source* [32, p. 3]. Key: *KII* Key Informant Interview; *FGD* Focus Group Discussions; *IDI* In-depth Interviews; *WASH* Water Sanitation and Hygiene; *UNICEF* United Nations International Children's Emergency Fund

The Socio-Ecological Framework (SEF) for MHM was used to identify and analyse the data used in this review [32]. This Framework was developed by UNICEF and Emory University to guide researchers globally to generate findings about factors that impact MHM. The Framework is intended to guide study design, with the five key factors; biological, personal, interpersonal, environmental and societal factors) considered relevant and useful to evaluate existing literature and reports on experiences about MHM (Fig. 2). The five factors of the SEF were used to analyse, identify, extract and tabulate factors affecting menstrual health from the 11 included studies on a spreadsheet, as shown in Tables 2 and 3. Publication year, authors name, country and study setting and the study aims were also extracted.

**Table 2:** Included literature synthesised using Socio-Ecological Framework

Publications (a)	Country & study setting	Study aim/area of enquiry	Biological factors	Personal factors	Interpersonal factors	Environmental factors	Societal factors	Summary of evidence
Clauson 2012 [42]	Fiji Village setting (Kadavu Island)	To understand how urbanisation affects way menstruation is treated in Fijian communities	No evidence	Girls aware of menstruation & menarche at pre-menarche. Supported by mothers, grandmothers & aunties. Girls learn about SRH at menarche ceremony	Girls learn about menstruation from village nurses & at schools. Learning in the presence of boys in schools is challenging. Teasing results from shame and embarrassment	No evidence	Evidence of cultural practices. Menarche ceremonies exists but rarely observed- religious influence & living away from home. Menarche ceremonies prepare girls for womanhood. Restrictive practices not evident	Menarche ceremonies used to prepare girls for womanhood. Education also reinforces pre-menarcheal learning No evidence of restrictive practices
Fitzgerald 1990 [35]	Western Samoa: Rural village (Savai); American Samoa: semi-urban villages, (Tutuila); Hawaiian Samoa: urban context, (Honolulu)	To see if culture change results in a change in menstrual experience	Stoic about menstrual pain but likely to seek medical attention. Behavioural changes linked to PMS leading to decreased activity, increased sleepiness	Evidence of females paying extra attention to personal hygiene & proper disposal of menstrual materials	Menstruation-viewed as normal, natural & private affair for women. Public evidence of sexual behaviour for unmarried women is a cause of shame to individuals and family	No evidence	Concepts of menstrual pollution & taboo-not evident; casual conversation about menstruation evident between intimates; believe-pre-menarcheal coitus is pre-requisite for menarche. Belief- menstruation is evidence of sexually active status	Cultural factors play significant role in the recognition, evaluation, and expression of menstrual symptoms. No evidence of menstrual pollution but increased attention to menstrual hygiene
Francois et al. 2017 [36]	Fiji School settings in Ba, Lautoka & Ra; rural, urban and peri-urban schools	To assess menstrual related challenges in schools	Evidence of menstrual pain and physical symptoms-tiredness, dizziness, causing exclusion from social & sporting activities in schools. Feel unprepared to manage symptoms	Lack menstruation knowledge; evidence of some knowledge gap; common knowledge source-mothers. Staining clothes cause fear, shame & lack of class concentration; staining leads to teasing	Evidence of learning from school, but teaching menstruation in presence of boys’ presence leads to shame, discomfort. Repeated teasing from boys prevents girls from seeking support	Evidence of adequate WASH facilities meeting standards. However, facilities lack privacy, security and sanitary pads; evidence shows challenges relating to inconsistent availability of materials^a^-due to frequent cyclones & floods	Cultural taboos & norms leads to lack of menstrual information. Evidence of discomfort learning about menstruation in same class with boys; cultural taboo causes discomfort for male teachers to teach menstruation topics. Some evidence of restrictive practices	Girls face multiple MHH challenges due to lack of knowledge & unpreparedness; menstrual pain; fear/embarrassment of staining clothes; teasing; lack of WASH facilities. Impacts on school attendance and participation
Hugget and Natoli 2017 [37]	Fiji School settings	Explore challenges experienced by women & girls managing menstruation & whether these challenges affect participation in school, work & community engagement	Many girls have pre-menarcheal awareness prior to menarche	Evidence of access to education about MHH, however gaps exists-monthly cycle; disabled females often excluded access to menstrual education; generational gap exist –older women uneducated. Menstruation restricts social activities	Menarche viewed-transition to womanhood; Women in workplaces & market vendors face challenges managing menstruation- single public sanitary facility. Mothers- information source but lack support. Teasing affects school attendance	Evidence of high standards of WASH facilities in some schools, workplaces and public places; some WASH facilities lack soap, toilet tissues, and sanitary disposal facilities. Evidence of some facilities being locked, unclean, & requiring user fees	Menstrual taboo-less strict, however levels of secrecy & discretion exist but vary according to religion and cultural background and prevailing attitudes & beliefs between Fiji’s two main ethnic groups: *i-Taukei* & Indo-Fijians; reaching menarche is celebrated event; strong restrictive practices exist with Indo-Fijians	Women face multiple challenges impacting on MHH. These include lack knowledge and unpreparedness; restrictive practices; menstrual materials and WASH facilities
Jenkins 1994 [38]	PNG Rural & urban communities	Acquire information on men’s & women’s sources & levels of knowledge of pregnancy, childbirth, their customary beliefs & practices about reproduction, fertility control & experiences & attitudes towards childbirth	No evidence	Women reported having prior knowledge about menstruation before menarche	Sources of information were mothers, older women, school teachers, aunties and peers. Parents control emerging sexuality of children. Sexuality information passed at a time deemed right by parents. Evidence of menarcheal ceremonies to prepare girls for womanhood	Lacking safe method of disposing soiled materials	Evidence of cultural beliefs and secrecy around menstruation. Taboo around menstrual blood-believed to be dirty and harmful. Restrictive food and behavioural restrictive practices exist	Cultural beliefs and practices restricts pre-menarcheal awareness Parents control flow of sexuality information. Menarche ceremonies used to teach/prepare young girls for womanhood
Mohammed and Natoli, 2017 [40]	PNG, Fiji & Solomon Island (SI) Urban and rural settings	Describe menstruation-related attitudes and beliefs that contribute to restrictive practices in PNG, SI & Fiji; the impact of these restrictions on the lives of women and girls	No evidence	Evidence of lack of menstruation knowledge including pre-menarcheal knowledge. Lack of knowledge results from taboo and communication secrecy. Feeling of shame evident leads to social exclusion & impacts on young women’s education	Evidence of teasing, harassment, stigma and shame about menstruation compelling girls to be more cautious and secretive. More common in PNG and Solomon Island than in Fiji. Young women leave school due to teasing and harassment including lack of proper MHM facilities	Evidence of barriers to effective MHH such as poor WASH facilities. Condition worse in PNG and SI than Fiji. Womens’ ability to effectively manage menstruation is limited by poor WASH facilities	Evidence of socio-cultural & religious beliefs & attitudes leads to behavioural restrictions of women. Restrictions impact on their ability to effectively manage MHH with dignity and fully participate in school, work, and broader community life. Belief that menstruation is dirty, menstruation and menstrual blood brings bad luck to men and boys, and menstruation related secrecy and shame exists. Evidence of menstruation and health-related beliefs	Restrictive practices are common in PNG and SI compared to Fiji. Restrictive practices common in rural than urban areas. Some restrictive practices were perceived desirable and driven by women themselves
Mohammed and Natoli 2017 [40]	PNG Urban and rural settings	Understand how women in PNG manage menstruation & explore barriers & challenges experienced by women in managing menstruation	No evidence	Girls lack comprehensive knowledge of menstruation- unprepared for menarche leading to shame. Evidence of teasing by boys leading to shame and embarrassment	Mothers, other female relatives, friends and female teachers are important source of information and support. Yet many lack understanding of menstruation and MHM	WASH facilities in schools and workplaces rarely meet needs for managing menstruation due to lack of water supply, non-functioning toilets, unclean and poorly maintained facilities and no disposal mechanism for used soiled menstrual material. Also lack of privacy, lack of secure places for washing and personal hygiene	Evidence of common beliefs and discriminatory attitudes around menstruation being dirty and unhealthy causing difficulty for women managing menstruation. Also impacts negatively on their emotional wellbeing. High level of secrecy is challenging and becomes an additional barrier to effective MHH	Women face multiple challenges that influence their ability to manage menstruation hygienically. These include lack knowledge and unpreparedness; restrictive practices; menstrual materials and WASH facilities
Natoli and Huggett 2016 [39]	Solomon Is. (SI) Urban and rural settings	Understand how women in SI manage menstruation & explore barriers & challenges experienced by women in managing menstruation	No evidence	Evidence of girls lacking menstrual knowledge and are unprepared for menarche leading to shame & embarrassment. Teasing from boys is evident leading to embarrassment	Support sources include mothers, other female relatives, friends and female teachers. However, many lack accurate and thorough understanding of menstruation and MHH	Schools, workplace’s lack WASH facilities for women to manage menstruation due to lack of water supply, non-functioning toilets, unclean & poorly maintained facilities. Lack of disposal mechanism for used materials Lack of privacy, lack of secure places for washing and personal hygiene-also evident	Beliefs &discriminatory attitudes around menstruation being dirty & unhealthy-evident, causing difficulty managing menstruation by women. These beliefs impacts on women’s emotional wellbeing. High level of secrecy is challenging and becomes an additional barrier to effective MHH	Challenges women face are multiple. These challenges influence women’s ability to manage menstruation hygienically. Challenges include lack knowledge and unpreparedness; restrictive & discriminatory practices; menstrual materials and WASH facilities
Sniekers 2005 [34]	Fiji Villages and urban settings (Suva and Nausori):	Gain knowledge of Fijian female gender identity through studying the menarcheal ceremony	No evidence	Evidence of feeling scared, shamed, embarrassed-private and personal	Evidence of learning from, schools teachers, mothers, aunties and grandmothers; focused learning about womanhood expectations, MHH,SRH occurs during menarcheal ceremonies	No evidence	Evidence of cultural secrecy & taboo of discussing sexuality; however, some evidence of menstrual blood is considered ‘mana’, harmful and sacred; menarche is celebrated event; no customs relating to restrictive practices; evidence of menarcheal ceremonies giving positive image but becoming rare	Although menarcheal ceremonies are becoming uncommon, female and womanhood identity is acquired through these ceremonies. Learning for girls also takes place in schools
UNICEF 2018 [41]	Kiribati South Tarawa, Abaiang & Abemam; rural & urban school settings	Explore the extent to which menstrual hygiene practices impact girl’s educational outcomes and development in Kiribati	Evidence of menstrual pain and loss of concentration from mood swings	Evidence of knowledge gap relating to menstruation & reproductive health in girls, including others: school aged-boys and mothers; girls lack knowledge to track onset of periods. Lack of knowledge in boys leads to curiosity, teasing and bullying of girls in schools. Teasing and bullying causes girls’ school absenteeism, shame & embarrassment	Teachers lack knowledge & awareness of MHH being part of curriculum; teachers under-resourced and undertrained to subjects and manage student’s expectations. Consequently, girls receive incomplete information regarding menstruation and SRH	. Evidence that safe sanitation is far below standard for menstruating girls; girls with disability face additional barriers in managing menstruation; evidence of school absenteeism & decreased school participation due to poor WASH conditions in schools to help manage menstruation. cloths, diapers & sanitary pads used to manage menstrual blood; product choice is determined by availability & accessibility to cash	. Evidence of traditional beliefs and practices in both urban and rural settings; beliefs that menstrual blood is taboo; restrictive practices; cultural beliefs causing restrictive communication amongst men and boys consequently leading to teasing and bullying of girls causing girls feeling scared & embarrassed; significance of culturally appropriate disposal method of soiled material is linked to cultural taboo around menstrual blood	Young women face multiple challenges managing menstruation due to lack of knowledge, teacher’s lack of knowledge, traditional restrictive practices, shame and secrecy, poor WASH facilities, bullying and teasing. This impacts on girls’ school attendance. Young women with a disability face additional challenges
Vallely et al. 2012 [33]	PNG Sexual Health Clinic in Port Moresby	To investigate intra-vaginal practices (IVP) and vaginal microbicide acceptability, and discuss implications of findings for future HIV prevention policy and research priorities	No evidence	Women perceive and support the view that menstrual blood is harmful Belief that the use of menstrual products blocks bad air from flowing inside of women’s body. Hence, women use ‘smoking & steaming’ practice to clean their birth canal	No evidence	Evidence that menstruating women cleanse vulva before sex with water, soap and vaginal inserts (crushed garlic) for improved genital hygiene and vaginal soap for vagina tightening. Customary ‘steaming’ practices and menstrual blood is absorbed using fragments of materials, cloths, newspapers, baby nappies, and sanitary towels	Evidence of traditional customs and norms relating to menstruation, beliefs and perceptions about menstrual blood. However some women admit having sex while menstruating	Diverse range of intra-vaginal practices were reported. Customary menstrual ‘steaming’ practices; use of fragments, cloths and newspapers to absorb menstrual blood were reported. Unprotected sex during menstruation was commonly reported

(a) Figure within parenthesis is reference number in reference list

**Table 3:** Quality assessment of included documents

Publication (a)	Article type (b)	Study design (c)	Was there a clear statement of the aim?	Is a (qualitative) method appropriate?	Was the research design appropriate to address the study aim?	Was recruitment strategy appropriate to research aim?	Was data collection method address research issue?	Has the relationship between researcher and participants been considered?	Have ethical issues been taken into consideration?	Was the data sufficiently rigorous?	Is there a clear statement of findings	Will result help locally? (valuable of the research)
			1	2	3	4	5	6	7	8	9	10
Clauson 2012 [42]	GL-RR	Original. research: Qual. design	Y	Y	Y	Y	Y	CT	Y	Y	Y	Y
Fitzgerald 1990 [35]	SA	Original research: Mix methods	Y	Y	Y	Y	Y	Y	Y	Y	Y	Y
Francois et al. 2017 [36]	GL-RR	Original research: Qual. Design	Y	Y	Y	Y	Y	N	Y	Y	Y	Y
Hugget and Natoli 2017 [37]	GL-RR	Original research: Qual. design	Y	Y	CT	Y	Y	Y	Y	Y	Y	Y
Jenkins 1994 [38]	SA-BC	Original research: Qual. design	Y	Y	Y	Y	Y	Y	Y	Y	Y	Y
Mohammed et al. 2018 [15]	SA	Original research: Qual. design	Y	Y	Y	Y	Y	Y	Y	Y	Y	Y
Mohammed and Natoli 2017 [40]	GL-RR	Original research: Qual. design	Y	Y	CT	CT	Y	Y	Y	Y	Y	Y
Natoli and Huggett 2016 [39]	GL-RR	Original research: Qual. design	Y	Y	Y	Y	Y	Y	Y	Y	Y	Y
Sniekers 2005 [34]	SA	Original research: Qual. design	Y	Y	CT	Y	CT	CT	N	CT	Y	Y
UNICEF 2018 [41]	GL-RR	Original research: Qual. Design	Y	Y	Y	CT	Y	N	Y	Y	Y	Y
Vallely et al. 2012 [33]	SA	Original research: Qual. design	Y	Y	Y	Y	Y	N	CT	Y	Y	Y

^a^*PMS* pre-menstrual syndrome

^b^Materials such as water, soap, toilet paper and sanitary pads

(a) Figure within parenthesis is reference number in reference list

(b) BC = Book chapter; GL = Grey Literature; SA = Scholarly article; RR = Research report; RA = Review article

(c) Qual = Qualitative design; Quant = Quantitative design; SRW = Systematic review; RW = Review (not systematic)

(d) Y = Yes; CT = Can’t tell; N = No (Abbreviation consistent with CASP tools)

Biological factors relate to age and intensity of menstrual cycle [32], while the personal factors relate to a girl’s knowledge, skills and beliefs. Interpersonal factors refer to the influence exerted by families, friends/peers and teachers. Community includes any member of the community with whom the menstruating woman associates. Environmental factors refer to menstrual issues relating to water, sanitation, hygiene and menstruation management material required to manage menstrual flow with dignity. Societal factors relate to policy, traditional cultural beliefs and social norms that potentially affect women’s ability to manage menstruation effectively. These five thematic areas were considered relevant to systematically analyse and generate evidence relevant to the research question of this scoping review.

## Results

The literature search resulted in 11 studies that met the inclusion criteria. Nine of these studies were conducted in Melanesian countries (Fiji, PNG and Solomon Islands), one Micronesian country (Kiribati), and one in Polynesian countries (Western Samoa and American Samoa). The 11 included studies comprised five peer-reviewed articles and six grey literature documents. One of these six is a book chapter [32]. This review found limited literature on factors affecting MHH amongst women in PICTs. Of the 11 included works, only four [15, 33–35] were peer-reviewed and published articles and out of the four scholarly articles, only one [15] is directly related to menstrual health and hygiene. This paucity of evidence clearly demonstrates a need for research on MHH in the PICTs.

The review identified and categorised factors affecting MHH under the five socio-ecological factors: biological, personal, interpersonal, environmental and societal. As presented in Table 2, all 11 studies reported elements of societal factors and personal factors; nine studies [15, 33, 34, 36–41] reported elements relating to environmental factors; ten studies [15, 34–42] found evidence relating to interpersonal factors and two studies [35, 36] presented evidence linking to biological factors. Data extracted from each of the 11 articles are categorised according to these factors (Table 2) and each is presented in turn.

### Biological factors

Only two studies found elements relating to biological factors (see Table 2). One study conducted in Western Samoa, American Samoa and Hawaiian Samoa reported behavioural changes such as dizziness and feeling lazy, sleepy and tired from pre-menstrual syndrome resulting in decreased activity [35]. A woman from Western Samoa said [35]; “*When I have it I don't do heavy work because my body feels weak (Western Samoan Woman).* While decreased activity was reported, the study did not report the implications of pre-menstrual syndrome for social participation, including school attendance for young women. The second study conducted in Fiji [36] reported evidence of menstrual pain and other physical symptoms such as feeling tired and dizzy. Unlike the Samoan study [35], the study in Fiji reported effect of menstrual symptoms on social and physical activities and young women were unprepared to manage these symptoms [36].

### Personal factors

All included studies reported evidence of personal factors (knowledge, attitudes, skills and beliefs) relating to pre-menarcheal awareness about menstruation and menstruation management skills. Ten included studies—all except the study with Samoan women [35]—reported young women lacking comprehensive pre-menarcheal knowledge about menstruation, feeling scared, embarrassed and confused at menarche, and lacked skills to effectively manage menstruation. However, evidence varied according to country and local contexts.

Many young girls generally lacked knowledge about menstruation before menstruation, the timing of menarche and Sexual Reproductive Health (SRH) knowledge. A study conducted in PNG found that many girls lacked comprehensive knowledge around menstruation and were unprepared at menarche, with some girls having misconception of what menstruation. One school girl said; *“When our mothers give birth to us, the waste remains in our bodies until the appropriate age when we menstruate, the waste blood comes out”* (FGD Girls in school; rural). A study in Kiribati [41, p. 16] reported a young school girl saying; *“menstruation is a sin from Eve who disobeyed God”* (Girl, Survey Responder).

The young girls exposed to information about SRH and menstruation were ready and knew how to manage menstruation. However, early exposure to menstruation knowledge varied between different countries and cultural context and were often limited. Compared to studies conducted in PNG [15, 40], Solomon Islands [15, 39] and Kiribati [41], the studies conducted in Fiji [15, 36, 37] reveal evidence of more access to education and information about menstruation and hygiene practices—commonly learnt at school before menarche. However, knowledge gaps relating to monthly menstruation cycles exist. Two studies conducted in Fiji reported menarcheal ceremonies, where young women are isolated and prepared by older female members of the family (mothers, aunties and grandmothers) for womanhood [34, 42].

Lack of knowledge and understanding about menstruation reportedly led to fear, embarrassment and confusion at menarche. These responses were commonly reported by young girls who were not educated about menstruation before menarche. Those who were educated had positive attitude at menarche by having the confidence of telling mothers, were prepared with menstruation materials and knew how to manage menstruation. Some girls in Fiji [36, 37] and Kiribati [41] also reported learning about menstruation in SRH classes in schools whilst in Solomon Islands [39] and PNG [40] most young women lacked pre-menarcheal knowledge about menstruation and were unprepared for menarche, subsequently experiencing feelings of fear, confusion, shame and embarrassment [39, 40]. In a study in PNG [40], a young female student related to her friend’s experience and said; *“She will be ashamed…sometimes she will be scared”* (Focus Group Discussion, Girls in school).

Young women lack knowledge and skills on how to effectively manage menstrual blood and safely dispose of soiled pads. The studies in PNG [15, 40] and Solomon Islands [15, 39] commonly found women lacked pre-menarcheal knowledge and the ability to manage menstruation. Young women with disability faced additional challenges because of lack of facilities to support disabled girls [36, 37, 39, 40]. A disabled women from Fiji reported [37, p. 23];

*“I find out that public facilities are not accessible for wheelchairs. Even though they try to make toilets very accessible for us, people with disability, especially wheelchair users. Because some of us cannot balance ourselves, you know using the toilet, they don’t have any railings” (In-depth Interview, Woman with disability, Fiji).*

Shame and embarrassment around MHH was found to restrict social and economic activities in studies conducted in Fiji, SI, PNG and Kiribati [15, 36, 37, 39–41]. Shame and embarrassment were also usually linked to teasing and bullying from males. Lack of knowledge about menstruation often resulted in teasing and bullying of young menstruating female students by male peers in schools. This evidence was reported in a study conducted in Kiribati [41] where knowledge gaps about menstruation and sexual reproductive health in school-aged boys about menstruation led to teasing and bullying. Lack of menstrual knowledge was commonly linked to taboo and secrecy resulting in limited communication, shame and embarrassment [15, 36, 37, 39, 41, 42] and is further explained under societal factors**.**

### Interpersonal factors

Interpersonal factors relating to relationships with families, teachers, peers and members of the community with whom the young women interact on a daily basis pose a variety of challenges. All 11 studies reported challenges relating to pre-menarcheal awareness and preparation (Table 2). Many young girls lacked awareness about menarche, timing of menarche and how to effectively manage MHH before the onset of menarche. Lack of knowledge, embarrassment and taboos were reported to be the common contributing factors to girl’s lack of awareness and pre-menarche preparation. Mothers are considered primary source of support for their daughters. Other sources include female relatives, girlfriends and teachers. However, the studies found that some mothers were often reluctant to talk with their daughters about menstruation because of shame, taboo and their own lack of appropriate SRH knowledge.

However, these experiences are contextual and vary between different countries, and cultural, educational and religious background. In a study in PNG [40, p. 15], a disabled woman reported that her mother did not advise her before the onset of menarche because of shame.

*“Really I have no idea what menstruation was all about, because it was my first time, my mother was ashamed and came and saw it, and she just told me you are … like other women… … it’s called period, monthly period and left”* (In-depth Interview Woman with a disability

Additionally, in Fiji, a health worker reported that mothers do not properly guide their children about menstruation [36, p. 13].

*“I don’t believe that they are being properly advised by their mothers. I was thankful to those health workers for properly explaining to the young girls at school about menstruating. Because I know that most mothers do not properly guide their children about their menstruation”* (Key Informant Interview, Health worker, Fiji).

In contrast, a study conducted in Fiji reported that menarche is celebrated and most i-Taukei Fijian (indigenous Fijians) women were able to talk to their daughters about menstruation and prepare them well by providing menstrual management products and taught them how to manage menstruation before the onset of menarche [42].

Menstruation topic was found to be a difficult topic to teach in schools. Most teachers lacked appropriate knowledge to effectively teach the subject and support young women in schools during menstruation. Female teachers who are also considered important source of information and support about menstruation sometimes lack accurate and comprehensive understanding of the issue and perpetuate misconceptions [39–41]. A study conducted in Solomon Islands [39, p. 13] reported that teachers were either too shameful to talk about menstruation or lacked accurate and comprehensive understanding to be able to teach the subject.

*“Now it [menstruation topic] is taught in Grade 3...but there is only cooperation for them to teach girls, but sometimes they don’t teach it because teachers have fear to teach it”* (Focus Group Discussion, Women in formal workplace, Solomon Islands.

*“One of the difficulties the teachers face- they have the knowledge to teach in class, but they need training in how to teach it”* (Key Informant Interview, Teacher, Solomon Island).

Male teachers often feel uncomfortable talking about menstruation, and need training and tools to assist them in this task [39–41]. A study in Kiribati found teachers lacked training to teach MHH and under-resourced to manage students’ expectations [41]. Although menstruation is taught in both rural and urban schools in PNG, teachers acknowledge lacking knowledge about menstruation, MHH and challenges associated with menstruation [40]. Studies in Fiji, PNG and SI [15, 36, 37, 39, 40] found teachers lacked comprehensive knowledge in teaching SRH topics, resulting in them feeling uncomfortable when talking about menstruation to young female students. This resulted in girls receiving inadequate information about SRH including menstruation. These challenges also lead to female students lacking knowledge about menstruation and leaving school due to the inability to manage their menstruation [15, 37, 39, 40, 43]. In Fiji, PNG, Solomon Islands and Kiribati, information on menstruation is predominantly taught in mixed girl’s and boys’ classes, limiting the depth and scope of knowledge that can be provided and increasing girls’ vulnerability to teasing [15, 36, 37, 39–41].

Menarche is viewed as an important transition to womanhood. Cultural process usually exist to support this transition of girls [34, 38, 42, 43]. Menarche ceremonies—the traditional ways of preparing and celebrating young women for womanhood were reported in Fiji [34, 42]. These studies reported that menarcheal ceremonies were an important juncture through which girls are informed directly about SRH and gender roles and responsibilities. The onset of menarche is viewed as signifying transition from childhood to womanhood and is also viewed as a “normal” bodily process [36, 37, 42]. While menstruation is considered taboo, the levels of secrecy and discretion vary according to religious and cultural background and prevailing attitudes and beliefs [36, 37]. For example for i-Taukei Fijians menarche is often viewed as a time for celebration while Fijians of Indian origins exclude girls from social activities such as accessing mosque or praying due to perception that menstrual blood is polluting [36, 37].

Girls are teased by some men and boys during menstruation. Seven studies [15, 36–41]. reported that teasing from males commonly led to girls feeling ashamed and embarrassed. Teasing was linked to lack of understanding about menstruation on the part of boys and men. A study in Kiribati [41, p. 24] reported that boys did not feel comfortable talking about menstruation because it is a taboo and therefore had limited understanding of why women and girls menstruate. *“The boys usually tease girls when they menstruate, because they haven’t experienced that. Like they can say negative comments like ‘dirty girl’. They can say stuff like ‘don’t sit near me as you are dirty’.”* (Female Teacher, Abaiang). The study also reported that the girls felt ashamed when they are mocked by fellow students—especially boys when menstrual blood leaks through their skirts [41].

### Environmental factors

Environmental factors related to WASH were found to exert a significant impact on the ability of young women to manage menstruation effectively and with dignity at home, in schools, at workplaces and public places [31]. Lack of these facilities impacted on school attendance, work, community participation and economic activities in studies conducted in Fiji [15, 36], SI [39], PNG [40] and Kiribati [41].

‘Adequate’ WASH facilities is defined as access to clean and female-only secluded toilets with running water, availability of toilet tissue, menstruation management materials (at reasonable cost) and sanitation facilities for disposing of soiled materials [31]. Comparing the adequacy of WASH facilities between countries, the studies in Fiji [15, 36, 37] consistently revealed that WASH facilities were better when compared to PNG [15, 40], Solomon Islands [15, 39] and Kiribati [41]. However, the WASH facilities were reported to be in poorer quality/condition in rural village settings compared to urban settings in all of these countries. Further comparison found that the urban squatter settlements in PNG, Solomon Islands and Kiribati have poorer WASH facilities than Fiji.

Working women reported leaving work and school girls reported leaving schools due to challenges in managing menstruation in workplaces [15, 37, 39, 40]. Study on menstruation conducted in PNG [40, p. 20] reported school girls being sent home by teachers because of lack of sanitary pads and facilities to manage menstruation.

*“[T]hose girls if they have their … period during the day, in the school exactly when they are in the class, then we don’t keep them back in the class, we just send them to go home, because there’s no pad...”* (Key Informant Interview, Teachers, PNG) [40, p. 20].

Cost, supply chain and material choices in managing menstruation were generally more challenging for girls and women in Solomon Islands compared to PNG, Fiji and Kiribati [36, 37, 39–41]. A study in PNG [40, p. 20] reported that; *“Most Papua New Guinea women cannot afford pads”* (Focus Group Discussion, Women in informal work, PNG). Access to cash played a bigger part in the determining the type of materials girls and women were able to acquire to help them manage menstruation. In all PICTs access to menstrual products was even more challenging for women living in rural areas compared to the urban areas [15].

### Societal factors

Cultural norms, beliefs and practices were found to affect women’s ability to manage menstruation effectively and with dignity. Traditional and cultural beliefs related to restrictive practices are linked to longstanding perceptions about the harmful nature of menstrual blood. These restrictive beliefs and practices were found to be common in studies conducted in PNG [15, 38, 40], Solomon Islands [15, 39] and Kiribati [41] and less common in Fiji [15, 36, 37]. However, within Fiji the practices varied according to religious and cultural background commonly among the main ethnic groups: *i-Taukei* and Indo-Fijians [34, 36, 37]. The i-Taukei people are indigenous Fijian while Indo-Fijians are predominantly Hindu or Muslims [36]. For example, for i-Taukei Fijians, menarche is celebrated and observed as a passage of girls to womanhood while Indo-Fijians tend to socially exclude girls when they are menstruating [36].

The traditional cultural beliefs and practices around menstruation vary between countries and context due to education, influence from religion and changes in traditional lifestyles due to Westernisation. The Last Taboo studies [15, 39, 40] conducted in PNG and SI, reported restrictive practices are more commonly practiced in rural than urban areas while in Kiribati [41], the traditional beliefs and practices are strong in both urban and rural settings. In two Fijian studies [34, 42] and one in Samoan countries [35] the restrictive practices were reported to be uncommon in both urban and rural areas. While there was little evidence of denial of cultural beliefs and practices around menstruation in Samoan countries (Western, American and Hawaiian), there was an assertion that culture relating to *‘*stoicism’ about menstrual pain was found to play a significant role in Western Samoa in the recognition and expression of menstrual symptoms [35].

Restrictive practices were found to affect women socially and psychologically including their ability to manage menstruation effectively. In a study conducted in Fiji, PNG and SI, Mohammed et al*.* (2018, pp.7–8) reported one of the women from PNG saying:

*“They are dirty and you know they have a … cultural belief. They think that you make the men … and the male sibling in the house … you know the food you touch makes them sick and they get older quicker and they don’t have the strength to work, you make them weak so … they won’t be … like physically active in doing men’s work … that’s the belief”.* (Key Informant Interview, Female Health Worker, PNG).

The significance of ceremonies marking menarche was reported in two anthropological studies [34, 42] conducted in Fiji. The same ceremony is alluded to by Jenkins (1994) in her report about PNG—describing it as ‘initiation’ or ‘menarche rituals’ [38, p. 27]. These authors reported the menarcheal ceremonies (or rituals) facilitate gender identity and preparation of girls for womanhood. Apart from preparation of womanhood, Jenkins (1994) explicitly explained that due to the belief that open communication about sexuality may pique curiosity (leading to earlier or greater experimentation with sex), menarcheal rituals or initiations are traditionally used by parents to control the flow of information about sexuality, sex and reproduction to young women [38, p. 27]. This ceremony typically involves mothers, grandmothers and aunties in teaching and advising young girls about SRH topics. However, this ceremony no longer occurs regularly due to changes in the traditional ways of life and education [34, 38, 42].

Some women continue to observe traditional beliefs around menstruation which exclude them from community, social participation and sexual activities. Menstruating women used customary menstrual steaming to clean blood that was “blocked” before sex, with the belief that steaming will allow free flow of blood and cleanse the vaginal area before sex [33]. Studies from PNG [15, 40], Kiribati [41] and SI [15, 39] found menstruating women were not allowed to cook and feed men or go near men because that can destroy men’s strength in warfare, gardening, fishing and hunting. The study in Samoa reported that menstruation is not considered a taboo concept or polluting and the restrictive belief systems and resulting changes in behaviour are individual choices [35]. While menstruating women paid attention to personal hygiene there was no elaborative evidence about MHM. However, men perceived that pre-menarcheal coitus is a pre-requisite for menarche to begin and coitus after childbirth for resumption of postpartum menstruation.

No study explicitly reported major policy issues such as policy review, budget provisions or minimum standards for addressing MHH practices.

Quality assessment of the 11 studies on Table 3 resulted in four studies meeting the criteria, five partially meeting the criteria as far as were reported and two did not meet all criteria as outlined in the CASP tools.

## Discussion

This review found that research on MHH is very limited in the PICTs, certainly beyond the anthropological literature about beliefs and practices around menarche and subsequent menstruation. This finding is consistent with a recent unpublished literature review on menstrual hygiene in the Pacific [44]. The lack of evidence on MHH demonstrates a lack of attention on addressing women’s SRH and in particular the menstrual issues in the Pacific [44]. This review found that research on MHH in the PICTs is largely externally driven and lacks critical indigenous and epistemological perspectives from Pacific Islanders. Furthermore, the research lacks approaches that empower participants with lived experience to have control over the research agenda, the process and actions to address their felt issues [45]. This view is important because menstrual issues affecting women in the Pacific are personal and ingrained into people’s way of life.

Despite different settings and populations, the synthesis of descriptive evidence from a few countries (PNG, SI, Fiji, Kiribati and Samoa) reflects common themes relating to MHH with manifestations that differ in response to context between and within countries. Using the Socio-Ecological Framework (SEF), this synthesis highlights multiple challenges faced by women in PICTs at menarche and throughout their reproductive life relating to MHH. These challenges have important implications for addressing sexual and reproductive health issues (specifically those relating to menstruation) in the PICTs. These implications are now discussed at individual, institutional and societal levels [46].

### Individual level

Pre-menarche girls generally lacked menstrual knowledge, and menstruation management skills—often leaving them unprepared for menstruation in both urban and rural areas. Mothers are considered a primary source of support for their daughters, however many of them lacked proper knowledge about menstruation and menstrual health. Menstrual taboos also limited mothers’ ability to freely discuss menstruation topics with their daughters. Consequently, mothers felt ashamed and lacked confidence to assist their daughters resulting in fear, shame and confusion. This finding is consistent to studies conducted in other LMIC countries where young women’s lack of knowledge about menstruation was related to their mother’s lack of proper knowledge about menstruation and menstrual taboos [46–49]. Women living in rural and isolated communities with limited access to education tend to face more difficulties [46–49]. Inability to effectively manage menstruation due to lack of skills and menstrual management materials were also reported in Sierra Leone leading to shame and embarrassment [43]. Restrictive practices can also potentially impact on a range of personal factors such as pre-menarcheal awareness and preparation, self-esteem and psychological wellbeing, and MHH practices [14, 50, 51].

### Institutional level

This review found lack of proper WASH facilities in schools, workplaces and communities in both urban and rural areas. This finding is consistent with many studies conducted on WASH and MHH in the urban and rural areas in LMICs [50–56]. Lack of WASH facilities impacts on women’s ability to effectively manage menstruation and to fully participate in community activities, education and work. This finding is also evident elsewhere [11, 12, 56–58], with a lack of toilet facilities in schools restricting girls from educational attendance [59], women’s employment, economic and religious activities [12].

Lack of WASH facilities often left women feeling ashamed and embarrassed. This review found girls absenting from schools as a result of lack of proper toilet and sanitation facilities. The structural challenges at school such as inadequate bathrooms also cause difficulties for girls to manage menstruation effectively in school [52–55]. Lack of access to basic and/or comprehensive WASH facilities are also linked to cultural perceptions around menstrual pollution and segregation of menstruating women from accessing common WASH facilities [19].

### Societal level

Findings from this review suggests that misconceptions and restrictive practices resulting from the social norms and beliefs around menstruation and menstrual blood is a determinant to women’s MHH, emotional and psychological wellbeing. Although the review found disparity in menstrual beliefs and practices between countries and between rural and urban areas, the findings suggest that these beliefs are ingrained in the cultural beliefs and perceptions of menstruation including menstrual blood [57–59] and may continue to influence MHH practices of women in the PICTs. Social norms and practices around menstruation are critical elements that influence women’s ability to manage MHH [48, 60–63].

Curiosity, shame and gender-bound secrecy were also found to influence behavioural practices such as bullying and teasing from male figures, and the inability of male teachers to effectively support female students during menstruation in schools. Inadequate knowledge of boys about SRH and menstruation have been found to help promote and perpetuate stigma, acts of teasing, and bullying of women during menstruation [47, 63–65].

Taboo and stigma against menstruating women leading to restricted SRH communication and menstruation, social and community participation, education and work, are considered forms of gender-based violence induced by patriarchal perceptions of menstrual blood [64, 66]. Stigma, shame and secrecy around menstruation are linked to menstrual taboos [7, 58, 67–69]. Myths and rumours about menstruation found in this review lead to fear, shame and self-isolation [41]. They were also found in studies conducted in India [70] and Ghana [71], leading to psychological distress in menstruating women.

The use of the Socio-Ecological Framework in this analysis has highlighted important challenges and multiple focus areas for interventions. A multi-level approach is required in order to facilitate and create a supportive environment for a positive menstruation experience.

## Recommendations

The majority of evidence related to the implications of traditional social and cultural norms, beliefs and practices on MHM. Policy related evidence relating to MHH was minimal; this may infer lack of evidence informing practice or a mere lack of consideration for gender-specific issues due to subordination of women, cultural taboos, shame and secrecy. Negative norms, beliefs and practices that condition the experiences of women and girls at menarche result from the predominant patriarchal social and cultural environment within PICTs. These norms, beliefs and practices cushion the interplay of negative perceptions and reactions that impede women’s ability to successfully manage MHM. Despite disparities in experience between countries, rural and urban contextual settings, following actions are recommended; planning to include males in SRH education programs in an effort to influence their perceptions about menstruation is paramount [8, 19, 36–41, 61]. WASH facilities should be considered beyond schools to include work environments, public spaces, rural and urban settings [19, 36–41]. WASH facilities should be an essential item in public resource planning linked to people’s movements and resettlements including following migration and disasters [8, 19, 36–41, 58, 61].

The menarcheal ceremonies provide an important alternative to SRH communication due to taboo and secrecy relating to menstruation and menstrual blood. The menarcheal ceremonies reported in Fijian are traditional cultural systems practices by i-Taukei Fijian group to prepare young women for womanhood. These practices assist young women to assume gendered roles and responsibilities required in the society they belong to [34]. These menarche ceremonies are also found among the Navajo tribe of America [72], and Maasai and Bemba tribes (Rhodesia) of Africa to prepare girls for womanhood [73, 74]. The elements of the menarche ceremonies could be explored further to inform the development of an alternative communication strategy for menstruation and SRH for girls in transition to womanhood. Developing menarcheal ceremonies into a contemporary learning hub for teaching SRH topics to adolescent females in the Pacific holds promise.

Country-specific research around menstruation is a necessary first step for PICTs and the author has gone onto conduct relevant MHH research in PNG [19]. Furthermore, because menstruation is deeply embedded in the social and cultural context of PICTs, research done by local researchers using socially and culturally situated approach is important to ensure the knowledge that is created is relevant. Leaders in PICTs must prioritise research that promotes an understanding of local socio-cultural norms around menstruation because menstrual experiences are contextual; given the diversity of social and cultural contexts in the PICTs, local contextual knowledge is paramount.

## Strengths and limitations

This is the first systematic scoping review to examine the social and cultural implications of women’s menstrual health in the Pacific. Consistent with indigenous epistemological standpoints, this review was led by a Pacific Island research scholar as part of her PhD studies. Conducted from an Australian university, this review had good access to peer-reviewed and grey literature and relevant worldwide websites. The author group included researchers conducting research on menstruation in PICTs and with international organizations.

Most research about MHM in PICTs (apart from anthropological studies) mostly commenced only a few years ago. Hence, the number of studies conducted thus far is insufficient to fully understand the factors influencing MHM and the broader implications of menstruation on the wellbeing of girls and women. Articles published in languages other than English were excluded, which may have excluded relevant literature from French-speaking Pacific islands and territories.

## Conclusion

Menstruating girls and women have the right to manage menstruation effectively and with dignity. It is evident from this review that societal, environmental, interpersonal and personal factors impact on the experiences of girls and women around menstruation. Of these four factors, socio-cultural norms, beliefs and practices appear to be extremely important underlying determinants that require locally-relevant action. Furthermore, because menstruation is deeply embedded in the social and cultural context of PICTs, research done by local researchers must use socially and culturally situated approaches to ensure the knowledge that is created is relevant. Consideration should be given to utilising socially and culturally relevant approaches to understand country-specific issues around menstruation.

## Data Availability

Not applicable.

## Abbreviations

MHH: Menstrual Health and Hygiene
WASH: Water, Sanitation and Hygiene
LMIC: Low to Middle Income Countries
PICT: Pacific Island Countries and Territories
SI: Solomon Island
PNG: Papua New Guinea
SEF: Socio Ecological Framework
SRH: Sexual Reproductive Health
